# Oral Candidiasis in COVID-19 Patients Under Critical Care Involving Non-albicans Candida Species Alongside Candida albicans

**DOI:** 10.7759/cureus.87949

**Published:** 2025-07-14

**Authors:** Saurabh Mitra, Priyanku Sharma, Kausik Ganguly

**Affiliations:** 1 Viral Research and Diagnostic Laboratory (VRDL) Department of Microbiology, Murshidabad Medical College and Hospital, Berhampore, IND; 2 Genetics, Independent Researcher, Kolkata, IND

**Keywords:** candida albicans, covid-19, critical care, non albicans candida, oral candidiasis

## Abstract

Introduction

People around the world faced the possibility of death during the COVID-19 outbreak, including in India in 2019. Coinfection of oral candidiasis-like symptoms was not uncommon among COVID-19-infected patients undergoing treatment with prolonged broad-spectrum antibiotics or corticosteroid therapy. The present study aims to characterize the prevalence profile of *Candida* species and their antifungal drug susceptibility patterns among patients with COVID-19.

Material and methods

A short-term, hospital-based observational study was conducted on patients with Reverse Transcription Polymerase Chain Reaction (RT-PCR)-confirmed COVID-19 infections admitted to the critical care unit of a medical college hospital who presented with oral candidiasis-like symptoms. Oral swab samples were collected and cultured on Sabouraud dextrose agar. Species identification was performed using the VITEK-2 automated system (Biomerieux, France).

Results

Among 544 patients diagnosed with COVID-19, 20 were microbiologically positive for oral candidiasis. *Candida albicans* was the predominant species, followed by *Candida glabrata*, *Candida tropicalis*, and *Candida kefyr*. Females (55%) exhibited a slightly higher susceptibility than males (45%). Antifungal susceptibility testing revealed one isolate with multidrug resistance and five additional isolates displaying resistant or intermediate drug sensitivity patterns. COVID-19-related immune suppression and associated overuse of antibiotics and corticosteroids contributed to an increased incidence of oral candidiasis due to opportunistic fungi, including *Candida albicans* and non-*albicans Candida* species.

Conclusion

The candidal species profile associated with oral candidiasis did not significantly alter during the COVID-19 period compared to pre-COVID-19 baselines.

Key message

There was no occurrence of emerging *Candida species* during the COVID-19 period.

## Introduction

During the COVID-19 pandemic, patients who were COVID-19 positive and admitted to the critical care unit (CCU) reported various oral complications [1]. Studies have reported changes in the microenvironment of the nasopharynx and oropharynx in COVID-19-infected patients [2] and co-infection with opportunistic fungal species (e.g*.,* *Candida species*) among those admitted to the CCU having had long-term corticosteroid therapy [3]. Due to overuse of antibiotics and corticosteroids and prolonged hospitalization, there seems to be a chance of alteration of the common candidal profile, which would result in oral candidiasis [4,5] and antifungal drug resistance [6]. Antifungal resistance in fungal pathogens poses a serious clinical challenge, particularly in immunocompromised patients. Fungi evade antifungal drugs through multiple mechanisms, including drug target alterations, as seen in *Candida species *with *ERG11 *mutations that reduce azole binding [7-9]. Overproduction of drug targets, efflux pumps such as ABC (ATP-binding cassette) and MFS (Major Facilitator Superfamily) transporters, and structural modifications that limit drug entry further contribute to resistance [10,11]. Additionally, some fungi bypass inhibited metabolic pathways, enzymatically degrade antifungal drugs, or secrete extracellular enzymes to neutralize them before they reach the cell. These mechanisms, observed in *Candida auris*, *Aspergillus fumigatus* and *Cryptococcus species*, highlight the urgent need for enhanced surveillance and novel antifungal strategies [9]. Therefore, the current study provides information on the Candidal profile, which could be responsible for oral candidiasis in COVID-19-infected patients with an antifungal susceptibility pattern.

## Materials and methods

Study design, collection, and microbial culture

This observational short-term study was conducted between the beginning of May 2021 and the end of November 2021. Approval for the study was obtained from the Institutional Ethical Committee at Murshidabad Medical College & Hospital, India (approval number: MSD/MCH/PR/2368/2020). Informed consent was received from all the participants in the study. COVID-19 positive (RT-PCR confirmed) patients aged between 18 and 80 years who were admitted to the CCU and later presented with oral candidiasis-like symptoms [12,13] were considered eligible for this study. Oral swab samples were collected using an aseptic method with sterile cotton swabs dampened with normal saline, following the ICMR guidelines [14] with proper protection by trained medical personnel. The saline-dampened swabs were then applied to the tongue, inner cheek lining (buccal mucosa), and the crevice between the lip and gums (labial sulcus) with swift and circular motions. After collection, the swabs were carefully sealed and transported in a sterile manner to the microbiology laboratory of the hospital and inoculated with Sabouraud dextrose agar and incubated at 37°C for 24 hours. Daily inspections were conducted on the plates to detect any yeast or yeast-like growth. Plates that showed no signs of mycological growth were discarded after 10 days of incubation and considered negative. The study adhered to the Declaration of Helsinki.

Identification of pathogenic agents

For the identification of the pathogenic yeast, the VITEK 2 YST card system (Biomerieux, France) was used following the manufacturer’s protocol.

Sequence retrieval and alignment

Genomic sequences corresponding to *Candida albicans*, non-*albicans Candida* (NAC) species (*C. tropicalis*, *C. glabrata*), *Aspergillus fumigatus*, *Rhizopus oryzae* and severe acute respiratory syndrome coronavirus 2 (SARS-CoV-2) isolate (OR539497.1) were retrieved from the NCBI GenBank database. The sequences were aligned using ClustalW in MEGA 11 software, applying a gap opening penalty of 15 and a gap extension penalty of 6.66 to ensure high alignment accuracy [15].

Antifungal susceptibility, minimal inhibitory concentration (MIC), and circos plot analysis

Following the manufacturer’s protocol of the VITEK 2 YST card system (Biomerieux, France), the anti-fungal susceptibility was assessed, and a Circos plot was generated. The VITEK 2 YST card system uses disposable cards containing wells with various antifungal agents (e.g., amphotericin B, fluconazole, voriconazole, flucytosine, and echinocandins (e.g., caspofungin, micafungin), covering the most clinically relevant drugs for yeast infections) at different concentrations. After inoculation with a standardized yeast suspension, the card is incubated and read automatically, providing both species identification and MIC values within approximately 15 hours. The peripheral part of the Circos plot generated using the MIC values represents the fungal isolates and the antifungal drugs. The internal connections depict the relationship between species and drug resistance and susceptibility patterns. Different colors indicate various levels of MICs and how the isolates are distributed among the antifungals. The width of the connections might represent the proportion of isolates with a specific MIC distribution.

Construction of the phylogenetic tree

The phylogenetic tree was constructed using the maximum likelihood method with the Tamura-Nei substitution model in MEGA 11. Model selection was based on Bayesian Information Criterion (BIC) scores to minimize bias. Bootstrap analysis was conducted with 1,000 replications, and evolutionary distances were calculated using the Kimura 2-parameter model [16]. Nodes with bootstrap values ≥ 0.70 were considered reliable, with values ≥ 0.85 indicating strong statistical support.

## Results

Inclusion of patients

Out of 554 patients admitted to the CCU between May 2021 and November 2021, 20 (3.61%) patients showed oral candidiasis-like symptoms, and oral samples were collected from them for further analyses.

Identification of pathogenic Candida variants

VITEK 2 YST card system identified *Candida albicans* (60%) as the most common isolate, followed by *Candida glabrata* (25%), *Candida tropicalis* (10%), and *Candida kefyr* (5%) as NAC species (see Table 1).

**Table 1:** Species isolated from culture positive cases (n=20)

Species identified	Number of isolates	Percentages
Candida albicans	12	60%
Candida glabrata	5	25%
Candida tropicalis	2	10%
Candida kefyr	1	5%

Distribution of gender among Candida-affected patients

Among the study sample (554), the male-to-female ratio was 9:11. During the study period, females showed a slightly increased tendency to *Candida-*related oral infection than male individuals (Table 2).

**Table 2:** Gender distribution of individuals affected by different species of Candida

Species	Male individual	Male percentage	Female individual	Female percentage
Candida albicans	5	25%	7	35%
Candida glabrata	2	10%	3	15%
Candida tropicalis	1	5%	1	5%
Candida kefyr	1	5%	0	0%
Total	9	45%	11	55%

Antifungal susceptibility test and minimal inhibitory concentration (MIC)

**Figure 1:**
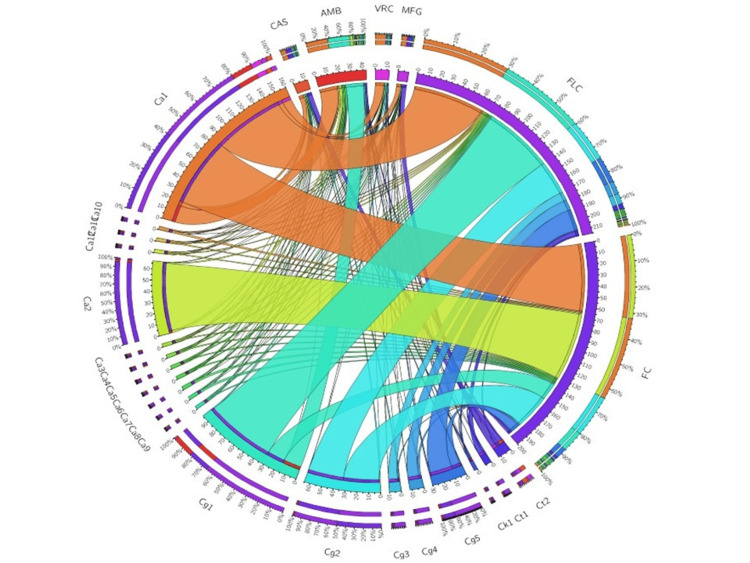
Circos plot displaying MIC distribution among 20 clinical isolates of Candida albicans (Ca) and non-albicans Candida (NAC) species Circos plot displaying MIC distribution among 20 clinical isolates of Candida albicans (Ca) and non-albicans Candida (NAC) species, Candida glabrata (Cg), Candida tropicalis (Ct), Candida kefyr (Ck) against six anti-fungal drugs: fluconazole (FLC), voriconazole (VRC), caspofungin (CAS), micafungin (MFG), amphotericin B (AMB), flucytosine (FC). In this Circos plot, the peripheral area shows isolates and antifungal drugs, and the internal area represents the relationship between them. FLC resistance: Several isolates, particularly *Candida albicans* (Ca1) and *Candida glabrata* (Cg1, Cg2), show high minimum inhibitory concentration (MIC) values (≥32 µg/mL), suggesting strong resistance. CAS and MFG effectiveness: Most isolates, regardless of species, display low MIC values (≤0.25 µg/mL) for CAS and MFG, except Ct2, which has elevated MICs (4 µg/mL). AMB and FC variability: AMB shows higher MIC values in some isolates (Ca1, Cg1, Cg5), raising concerns about possible resistance. FC resistance is particularly high in *C. albicans* (Ca1, Ca2) and *C. glabrata* (Cg1, Cg2, Cg5), with MIC values ranging from 8 to 64 µg/mL.

Antifungal susceptibility data for culture-positive isolates against six antifungals are detailed in Table 3. The minimal inhibitory concentration (MIC) ranges for *C albicans* and other non-*albicans Candida* species against fluconazole (FLC), voriconazole (VRC), caspofungin (CAS), micafungin (MFG), amphotericin B (AMB), and flucytosine (FC) are listed in Table 4. Among 12 *Candida albicans* isolates, one isolate (Ca1) (see Table 3) exhibited multidrug resistance and corresponding high MIC values. Another isolate (Ca2) showed resistance to FC (MIC >=64) and intermediate to AMB (MIC >=2). In the case of *Candida glabrata* isolates, Cg1 showed resistance to FLC (MIC>=64), AMB (MIC >= 16), and intermediate to FC (MIC >=16); and another isolate (Cg2) (Table 3) of *Candida glabrata* showed resistance to FC (MIC >=32). Of two *Candida tropicalis* isolates, one isolate (Ct1) (Table 3) displayed an intermediate MIC value to FLC (MIC>=4), and another one (Ct2) (Table 3) showed resistance to CAS and MFG (MIC >= 4, >= 4). The antifungal susceptibility testing revealed a wide MIC variation among *Candida albicans* and non-*albicans Candida* species, with notable resistance patterns. High MIC values for FLC and AMB were observed in some *C. albicans* and *C. glabrata* isolates (e.g., Ca1, Cg1), indicating potential resistance. CAS and MFG generally showed lower MIC values, suggesting better efficacy. The Circos plot (Figure 1) visually represents these MIC distributions, highlighting drug-isolate associations and resistance trends across the six antifungals.

**Table 3:** MIC distribution among culture positive isolates (Candida albicans and non albicans candida species) MIC=Minimum inhibitory concentration, Ca=*Candida albicans*, Cg= *Candida glabrata*, Ct= *Candida tropicalis*, Ck= *Candida kefyr*, FLC= Fluconazole, VRC=Variconazole, CAS=Caspofungin, MFG=Micafungin, AMB=AmphotericinB, FC=Flucytosine. Counts of resistant and intermediate strains are mentioned in bold and italic.

Isolates	FLC	VRC	CAS	MFG	AMB	FC
Ca1	64	8	4	4	16	64
Ca2	1	0.12	0.25	0.06	2	64
Ca3	1	0.12	0.25	0.06	0.5	1
Ca4	1	0.12	0.25	0.06	1	1
Ca5	1	0.12	0.25	0.06	1	1
Ca6	1	0.12	0.25	0.06	0.5	1
Ca7	1	0.12	0.25	0.06	0.25	1
Ca8	1	0.12	0.25	0.06	0.25	1
Ca9	1	0.12	0.25	0.06	0.5	1
Ca10	1	0.12	0.25	0.06	0.25	1
Ca11	1	0.12	0.25	0.06	1	1
Ca12	1	0.12	0.25	0.06	0.5	1
Cg1	64	1	0.25	0.06	16	16
Cg2	32	0.25	0.25	0.06	0.5	32
Cg3	8	0.12	0.25	0.06	0.5	1
Cg4	10	0.12	0.25	0.06	0.25	1
Cg5	20	1	0.25	0.06	0.5	8
Ct1	4	0.12	0.25	0.06	0.25	1
Ct2	1	0.12	4	4	1	2
Ck1	1	0.12	0.25	0.12	0.5	1

**Table 4:** MIC range for Candida albicans and non-albicans candida species MIC=Minimum inhibitory concentration, FLC= Fluconazole, VRC=Variconazole, CAS=Caspofungin, MFG=Micafungin, AMB=AmphotericinB, FC=Flucytosine.

Species	FLC	VRC	CAS	MFG	AMB	FC
Candida albicans	1-64	0.12-8	0.25-4	0.06-4	0.25-16	1-64
Candida glabrata	8-64	0.12-1	0.25	0.06	0.25-16	1-32
Candida tropicalis	1-4	0.12	0.25-4	0.06-4	0.25-1	1-2
Candida kefyr	1	0.12	0.25	0.12	0.5	1

Assessment of phylogenetic association, bootstrap analysis, and clade interpretation

**Figure 2:**
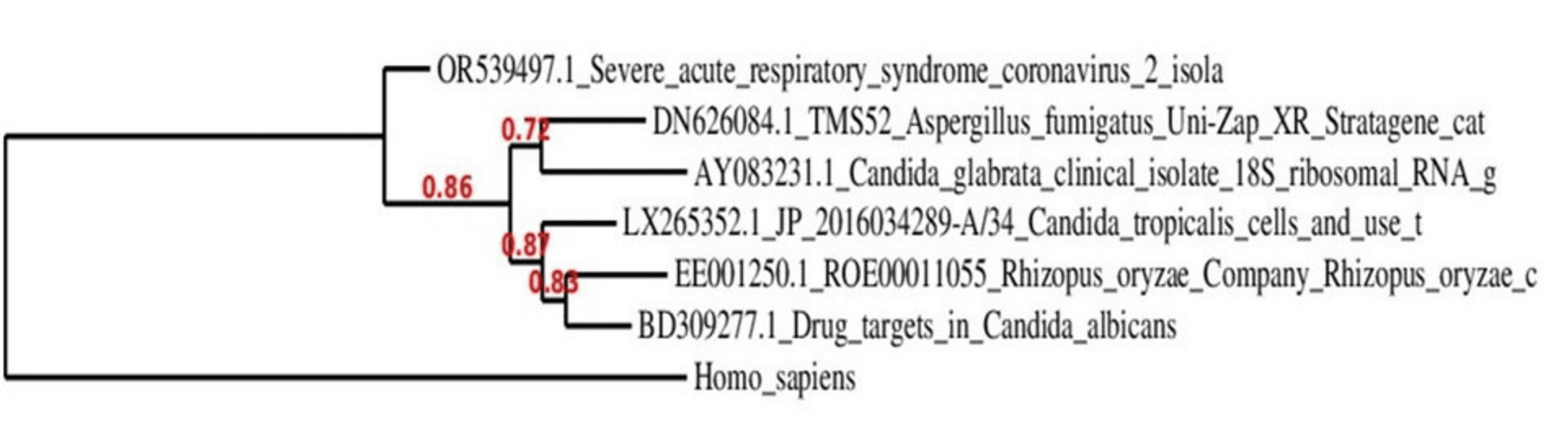
Phylogenetic relationships chart Phylogenetic relationships among severe acute respiratory syndrome coronavirus 2 (SARS-CoV-2), *Candida albicans*, non-albicans Candida (NAC) species, *Aspergillus fumigatus*, and *Rhizopus oryzae* (primary organism for mucormycosis). This analysis was conducted to rule out interspecies relationships between *Candida albicans* and NAC species with two other important opportunistic fungi (*Aspergillus fumigatus* and *Rhizopus oryzae*), which were active during the COVID-19 pandemic. In phylogram *Candida albicans*, *Candida tropicalis,* and *Rhizopus oryzae* made one clade, and *Candida glabrata* made another clade with *Aspergillus fumigatus*. These results indicate that interspecies variation is present in between *Candida albicans* and NAC species. The chart is prepared by the authors.

The phylogenetic tree (see Figure 2) revealed distinct evolutionary relationships. *Candida albicans* (BD309277.1) formed a clade with *Candida tropicalis* (LX263552.1) and *Rhizopus oryzae *(EE001250.1), supported by a bootstrap value of 0.85, suggesting a moderate evolutionary relationship. This finding aligns with prior studies indicating that *C. tropicalis* shares virulence traits and biofilm formation abilities similar to *C. albicans* [17]. Conversely, *Candida glabrata* (AY083231.1) grouped with *Aspergillus fumigatus* (DN620084.1), supported by a bootstrap value of 0.86, reinforcing previous reports that *C. glabrata* is phylogenetically closer to *Saccharomyces cerevisiae* than to *C. albicans* [18].

## Discussion

**Figure 3:**
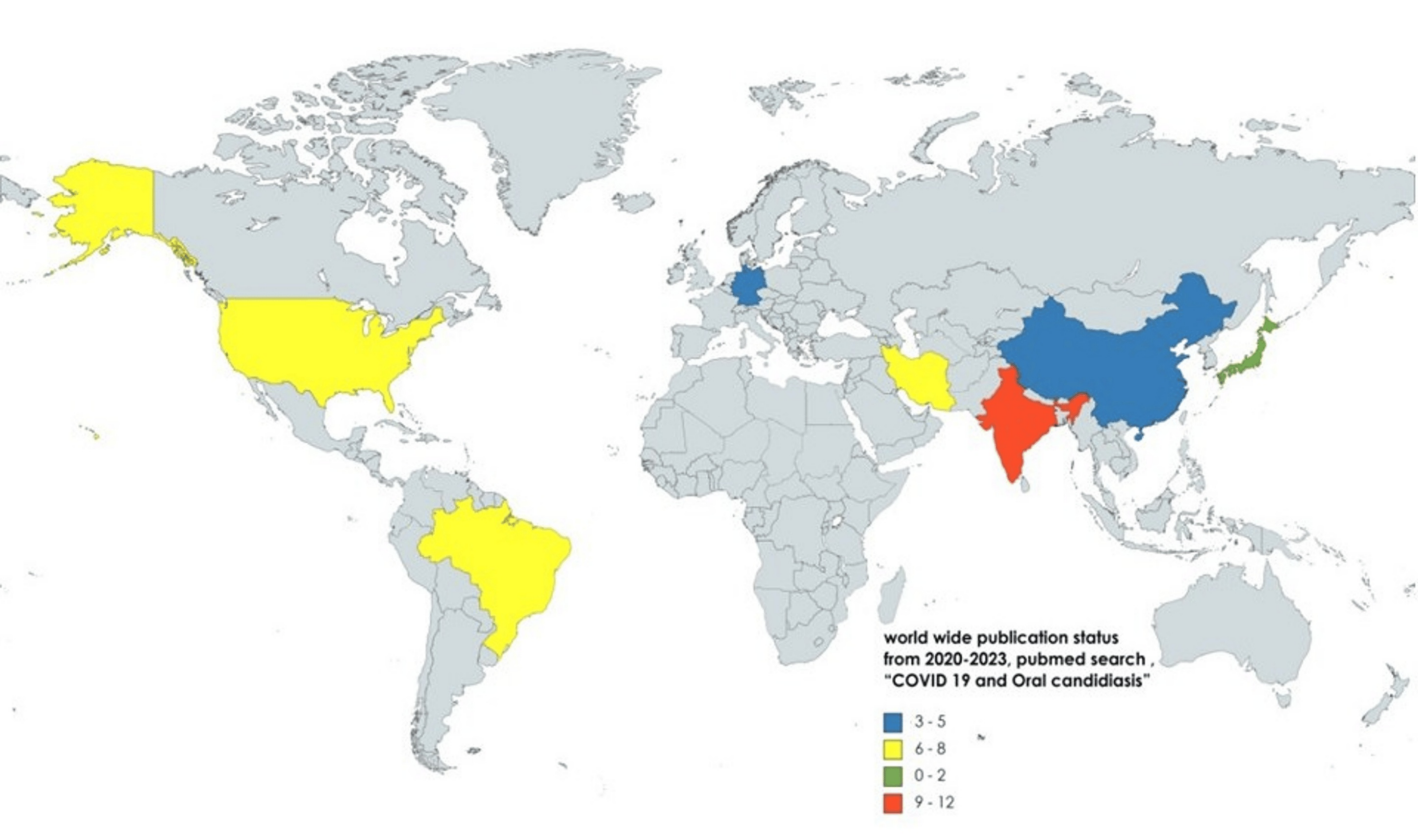
PubMed search (“COVID-19” and “Oral candidiasis”) showing worldwide publication status during the year 2020-2023. Illustration was created by Dr. Priyanku Sharma and Dr. Saurabh Mitra, based on the information available at MEDLINE database (PubMed search engine)

**Figure 4:**
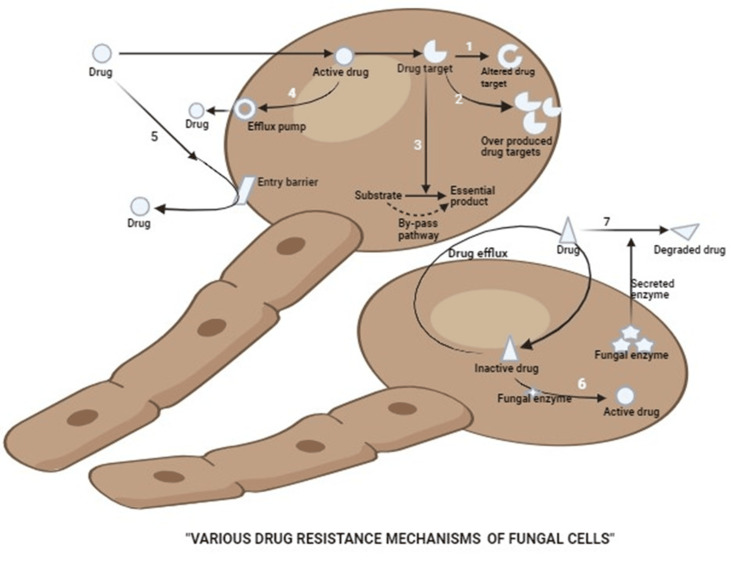
Drug resistance mechanisms in fungal cells The image is a schematic representation of seven types of mechanisms by which fungal pathogens evolve resistance to antifungal drugs. The illustration was created by the authors Priyanku Sharma and Saurabh Mitra, based on the information available in the literature [8,9].

Typically, Candida species are considered opportunistic fungi for human hosts, which is why oral candidiasis is relatively uncommon in a healthy human population. But in some instances, such as in chronic diabetes patients, cancer patients, HIV-positive individuals, denture users, smokers, and long-term users of antibiotics or corticosteroids, the occurrence of oral candidiasis is increased. In immune-compromised patients, the chance of occurrence of oral candidiasis is increased. A PubMed search identified 68 research articles on oral candidiasis in conjunction with COVID-19 infection between 2020 and 2023, the peak years of COVID-19 cases were observed (see Figure 3). The limited options available as anti-fungal therapy, ever-increasing antifungal resistance, and emergence of multidrug-resistant fungi through multiple mechanisms (see Figure 4) have multiplied the magnitude of complexities in the management of fungal infections like candidiasis.

In this study, the authors aimed to assess the identification of pathogenic agents responsible for oral candidiasis among 20 (3.67%) out of 544 COVID-19 positive patients, who were admitted to the CCU between May 2021 and November 2021. The predominant species was found to be *Candida albicans,* followed by *Candida glabrata*, *Candida tropicalis,* and *Candida kefyr*. This indicated that the normal profile of *Candida* species causing oral candidiasis [4,5] did not change during the pandemic. During the study period, females (55%) showed a slightly greater tendency toward oral candidiasis than males (45%).

Additionally, phylogenetic analysis plays a crucial role in understanding the evolutionary relationships among fungal pathogens, particularly in the context of antifungal resistance and pathogenicity. Given the rise of fungal co-infections during the COVID-19 pandemic, comparative genomic studies have become essential for evaluating interspecies variation and potential implications for treatment strategies [10,11]. In this study, the phylogenetic relationship between *Candida albicans*, NAC species, *Aspergillus fumigatus*, and *Rhizopus oryzae* was examined to assess genetic divergence and evolutionary clustering patterns. The observed inter-species variation is consistent with the known differences in antifungal resistance mechanisms (see Figure 4).

*Candida glabrata* exhibits intrinsic azole resistance due to overexpression of efflux pumps, unlike *C. albicans*, which is more susceptible to azole therapy but develops resistance through *ERG11* mutations and biofilm-associated mechanisms [18,19]. The unexpected clustering of *Rhizopus oryzae* with *C. albicans* and *C. tropicalis* may indicate potential shared pathogenic traits, as mucormycosis was frequently reported as a secondary infection in COVID-19 patients receiving prolonged corticosteroid therapy [20]. The relatively high bootstrap values (≥0.85) indicate reliable clustering patterns, confirming that *C. albicans* and NAC species exhibit significant phylogenetic divergence.

The above findings emphasize the need for species-specific treatment strategies, particularly for NAC infections, which are increasingly recognized as emerging threats because of their variable antifungal susceptibility profiles. Bootstrap support values were used to validate the evolutionary relationships among species. Clades with bootstrap values ≥ 0.85 were considered highly reliable, whereas those between 0.70 and 0.84 were moderately supported. *Candida albicans* grouped with *C. tropicalis* and *Rhizopus oryzae* (0.85), suggesting a moderate evolutionary link, whereas *C. glabrata* formed a separate clade with *A. fumigatus* (0.86), reinforcing its genetic distinction from *C. albicans*. The evolutionary placement of *Rhizopus oryzae* within the *Candida* cluster warrants further investigation to determine potential similarities in host adaptation and pathogenicity [21]. The results underscore the genetic and functional divergence between *C. albicans* and NAC species, with significant implications for antifungal resistance and clinical management. Understanding these evolutionary relationships is crucial for developing targeted therapies, particularly given the rising incidence of multidrug-resistant NAC infections in immunocompromised patients [22]. Moreover, COVID-19 played its role in immune dysregulation, characterized by lymphopenia, reduced CD4+/CD8+ T cells, and impaired neutrophil and macrophage function [23]. These changes weaken host defenses against opportunistic pathogens like *Candida species*. Additionally, the cytokine storm observed in severe COVID-19 led to tissue damage and an immunosuppressive rebound, creating a favorable environment for fungal invasion [24]. The use of immunomodulators such as corticosteroids further dampened cellular immunity, increasing susceptibility to mucosal and invasive candidiasis, leading to *Candida *overgrowth, translocation, resulting in altered oral and gut microbiota [25].

During the COVID-19 pandemic, various lifestyle alterations, viz. prolonged mask usage, reduced physical activity, poor dietary habits, and increased use of antibiotics or corticosteroids, have been linked to disruptions in the host microbiota and immune dysregulation, predisposing individuals to opportunistic fungal infections [26,27]. Moreover, oral hygiene was often neglected during this period due to limited access to dental care or patient self-neglect, further increasing the risk of oral colonization by *Candida* species [28]. In hospitalized or mechanically ventilated patients, inadequate oral care has been associated with enhanced biofilm formation and mucosal colonization by *Candida*, contributing to the development of both local and systemic co-infections [29]. Thus, the current study tried to delineate characteristics of oral candidiasis among COVID-19-positive patients.

## Conclusions

The stability of the *Candida* species profile in oral candidiasis during the COVID-19 period, despite widespread antibiotic and corticosteroid usage, suggests the absence of any emerging, previously unknown *Candida* outbreak. This suggests that prevalent non-*albicans Candida* (NAC) species, in conjunction with *Candida albicans*, remained active in the etiology of oral candidiasis among COVID-19-positive patients admitted to the critical care unit.

## References

[1] Hocková B, Riad A, Valky J (2021). Oral complications of ICU patients with COVID-19: case-series and review of two hundred ten cases. J Clin Med.

[2] Mitra S, Sharma P, Mandal C (2023). Study of micro-environmental change of nasopharynx and oropharynx in Covid infected patients and its effect on other known commensals and pathogenic bacteria. Int J Sci Res.

[3] Salehi M, Ahmadikia K, Mahmoudi S (2020). Oropharyngeal candidiasis in hospitalised COVID-19 patients from Iran: species identification and antifungal susceptibility pattern. Mycoses.

[4] Aslani N, Janbabaei G, Abastabar M (2018). Identification of uncommon oral yeasts from cancer patients by MALDI-TOF mass spectrometry. BMC Infect Dis.

[5] Lewis MA, Williams DW (2017). Diagnosis and management of oral candidosis. Br Dent J.

[6] Silva S, Negri M, Henriques M, Oliveira R, Williams DW, Azeredo J (2012). Candida glabrata, Candida parapsilosis and Candida tropicalis: biology, epidemiology, pathogenicity and antifungal resistance. FEMS Microbiol Rev.

[7] Perlin DS, Rautemaa-Richardson R, Alastruey-Izquierdo A (2017). The global problem of antifungal resistance: prevalence, mechanisms, and management. Lancet Infect Dis.

[8] Revie NM, Iyer KR, Robbins N, Cowen LE (2018). Antifungal drug resistance: evolution, mechanisms and impact. Curr Opin Microbiol.

[9] Cowen LE, Sanglard D, Howard SJ, Rogers PD, Perlin DS (2014). Mechanisms of antifungal drug resistance. Cold Spring Harb Perspect Med.

[10] Babamahmoodi F, Rezai MS, Ahangarkani F (2022). Multiple Candida strains causing oral infection in COVID-19 patients under corticosteroids and antibiotic therapy: an observational study. Front Cell Infect Microbiol.

[11] Vinayagamoorthy K, Pentapati KC, Prakash H (2022). Prevalence, risk factors, treatment and outcome of multidrug resistance Candida auris infections in Coronavirus disease (COVID-19) patients: a systematic review. Mycoses.

[12] Millsop JW, Fazel N (2016). Oral candidiasis. Clin Dermatol.

[13] Akpan A, Morgan R (2002). Oral candidiasis. Postgrad Med J.

[14] (2025). Laboratory testing for coronavirus disease 2019 (‎COVID-19)‎ in suspected human cases: interim guidance. https://iris.who.int/handle/10665/331329.

[15] Kumar S, Stecher G, Li M, Knyaz C, Tamura K (2018). MEGA X: molecular evolutionary genetics analysis across computing platforms. Mol Biol Evol.

[16] Tamura K, Nei M, Kumar S (2004). Prospects for inferring very large phylogenies by using the neighbor-joining method. Proc Natl Acad Sci U S A.

[17] Pristov KE, Ghannoum MA (2019). Resistance of Candida to azoles and echinocandins worldwide. Clin Microbiol Infect.

[18] Gabaldón T, Naranjo-Ortíz MA, Marcet-Houben M (2016). Evolutionary genomics of yeast pathogens in the Saccharomycotina. FEMS Yeast Res.

[19] Morace G, Perdoni F, Borghi E (2014). Antifungal drug resistance in Candida species. J Glob Antimicrob Resist.

[20] Aranjani JM, Manuel A, Abdul Razack HI, Mathew ST (2021). COVID-19-associated mucormycosis: evidence-based critical review of an emerging infection burden during the pandemic's second wave in India. PLoS Negl Trop Dis.

[21] Vitale RG, de Hoog GS, Schwarz P (2012). Antifungal susceptibility and phylogeny of opportunistic members of the order mucorales. J Clin Microbiol.

[22] Papon N, Courdavault V, Clastre M, Bennett RJ (2013). Emerging and emerged pathogenic Candida species: beyond the Candida albicans paradigm. PLoS Pathog.

[23] Giamarellos-Bourboulis EJ, Netea MG, Rovina N (2020). Complex immune dysregulation in COVID-19 patients with severe respiratory failure. Cell Host Microbe.

[24] Chen G, Wu D, Guo W (2020). Clinical and immunological features of severe and moderate coronavirus disease 2019. J Clin Invest.

[25] Hoenigl M, Seidel D, Sprute R (2022). COVID-19-associated fungal infections. Nat Microbiol.

[26] Salehi M, Ahmadikia K, Badali H, Khodavaisy S (2020). Opportunistic fungal infections in the epidemic area of COVID-19: a clinical and diagnostic perspective from Iran. Mycopathologia.

[27] Rawson TM, Moore LS, Zhu N (2020). Bacterial and fungal coinfection in individuals with coronavirus: a rapid review to support COVID-19 antimicrobial prescribing. Clin Infect Dis.

[28] Alsulami M, Kattan W, Alsamadani L, Alahmari G, Al Juhani W, Almabadi M (2023). An outlook on dental practices to avoid the oral transmission of COVID-19. Microorganisms.

[29] Gangneux JP, Bougnoux ME, Dannaoui E, Cornet M, Zahar JR (2020). Invasive fungal diseases during COVID-19: we should be prepared. J Mycol Med.

